# Supplementary material for: Quantitative analysis of tumour spheroid structure
Source: eLife. 2021 Nov 29;10:e73020. doi: 10.7554/eLife.73020 (PMC8741212; doi:10.7554/eLife.73020)
Supplement: Supplementary file 2. [file elife-73020-supp2.pdf]

Supplementary file 2

Subset of spheroid cross-sectional confocal images. We collect data on enough spheroids to ensure at least 10 for each initial seeding density and observation day (20 for comparing steady-state structure) are available. We then randomly subsample to ensure a consistent number of spheroids are analysed for each initial seeding density and observation day. In Fig. 1 to Fig. 6 we show a random subset of 10 spheroids from the complete data set for each condition, from days 7 to day 21 (WM983b) and day 24 (WM793b). Raw data (complete and the subset) are available on GitHub*.

WM983b (seeded with 2500 cells)

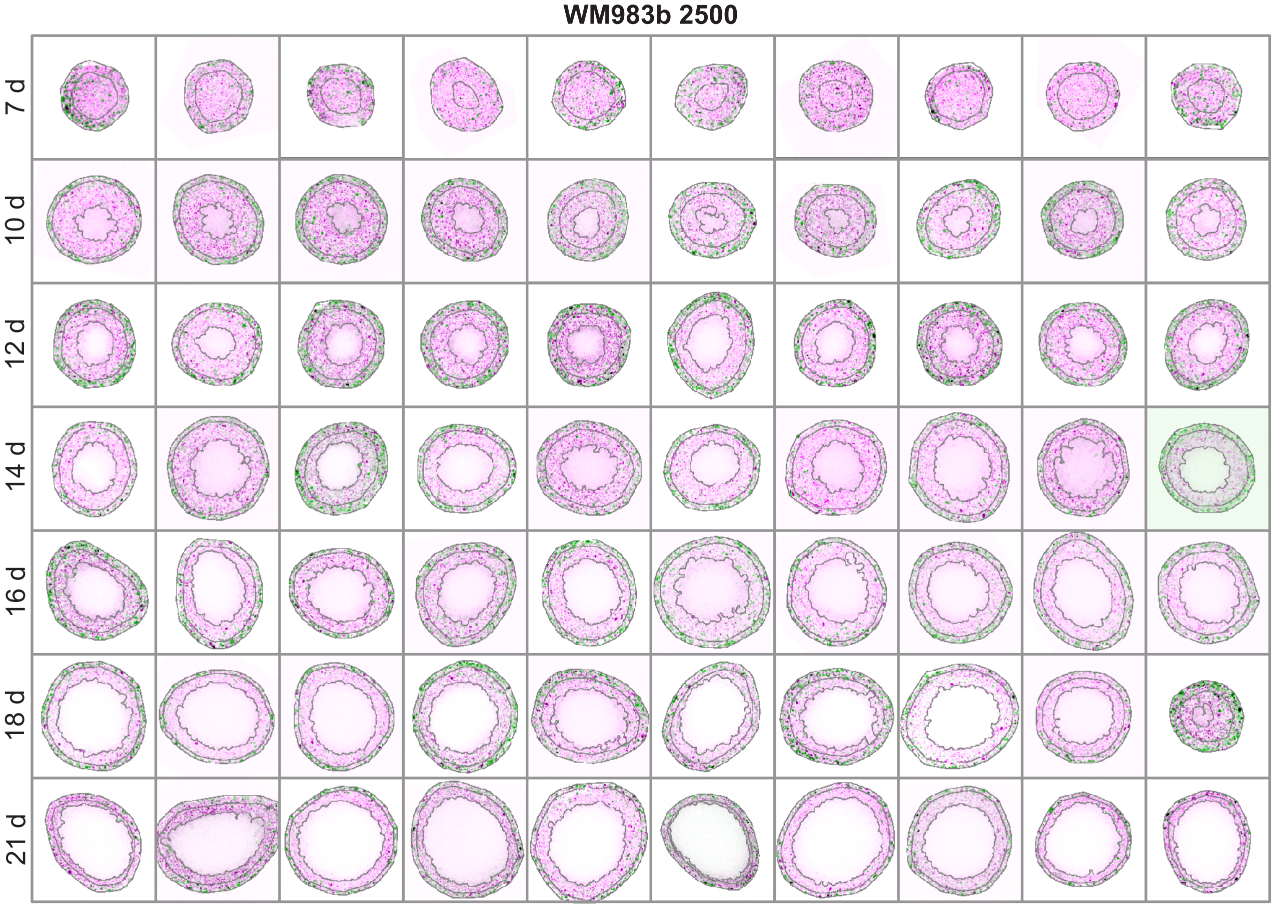

Figure 1

*github.com/ap-browning/Spheroids

WM983b (seeded with 5000 cells)

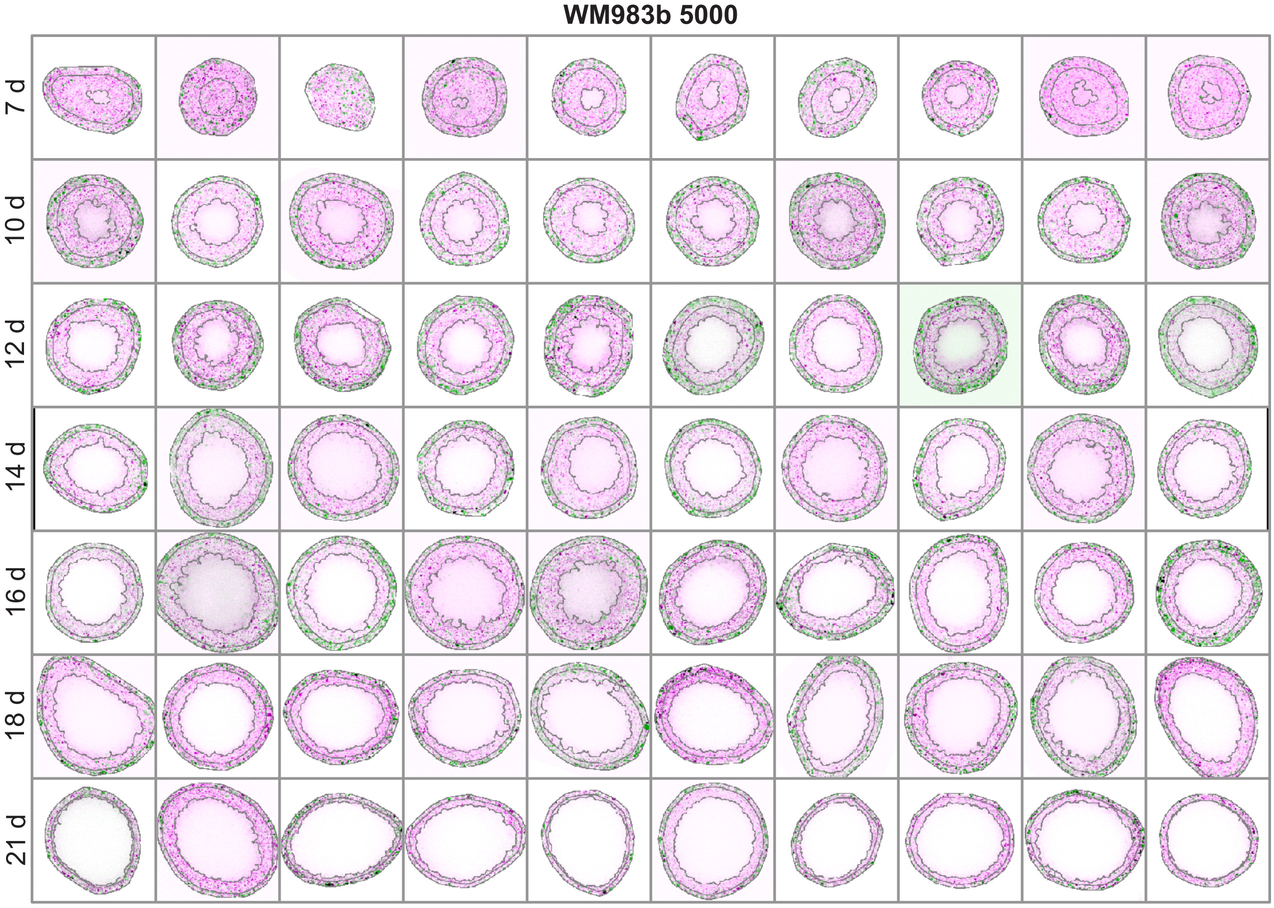

Figure 2

WM983b (seeded with 10000 cells)

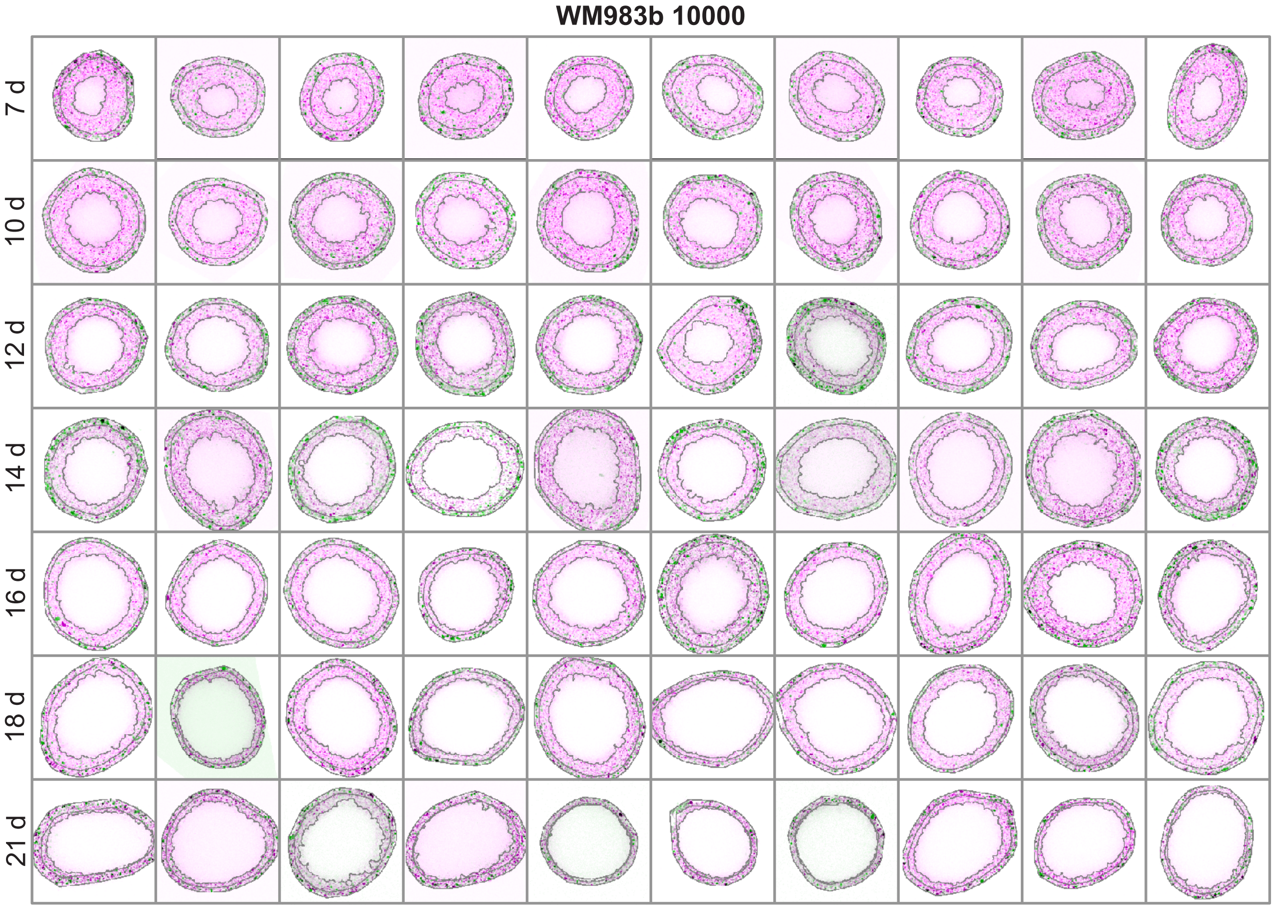

Figure 3

WM793b (seeded with 2500 cells)

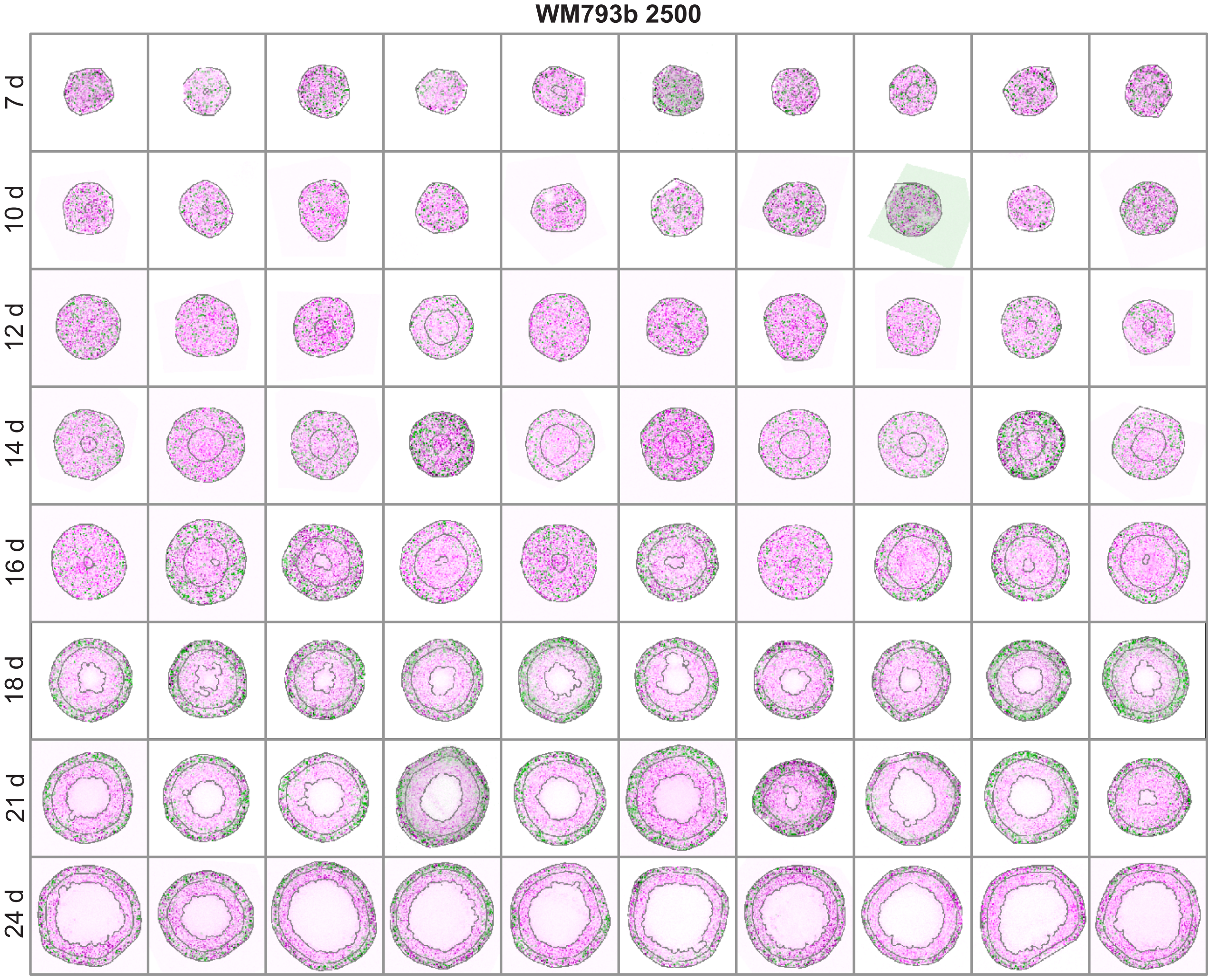

Figure 4

WM793b (seeded with 5000 cells)

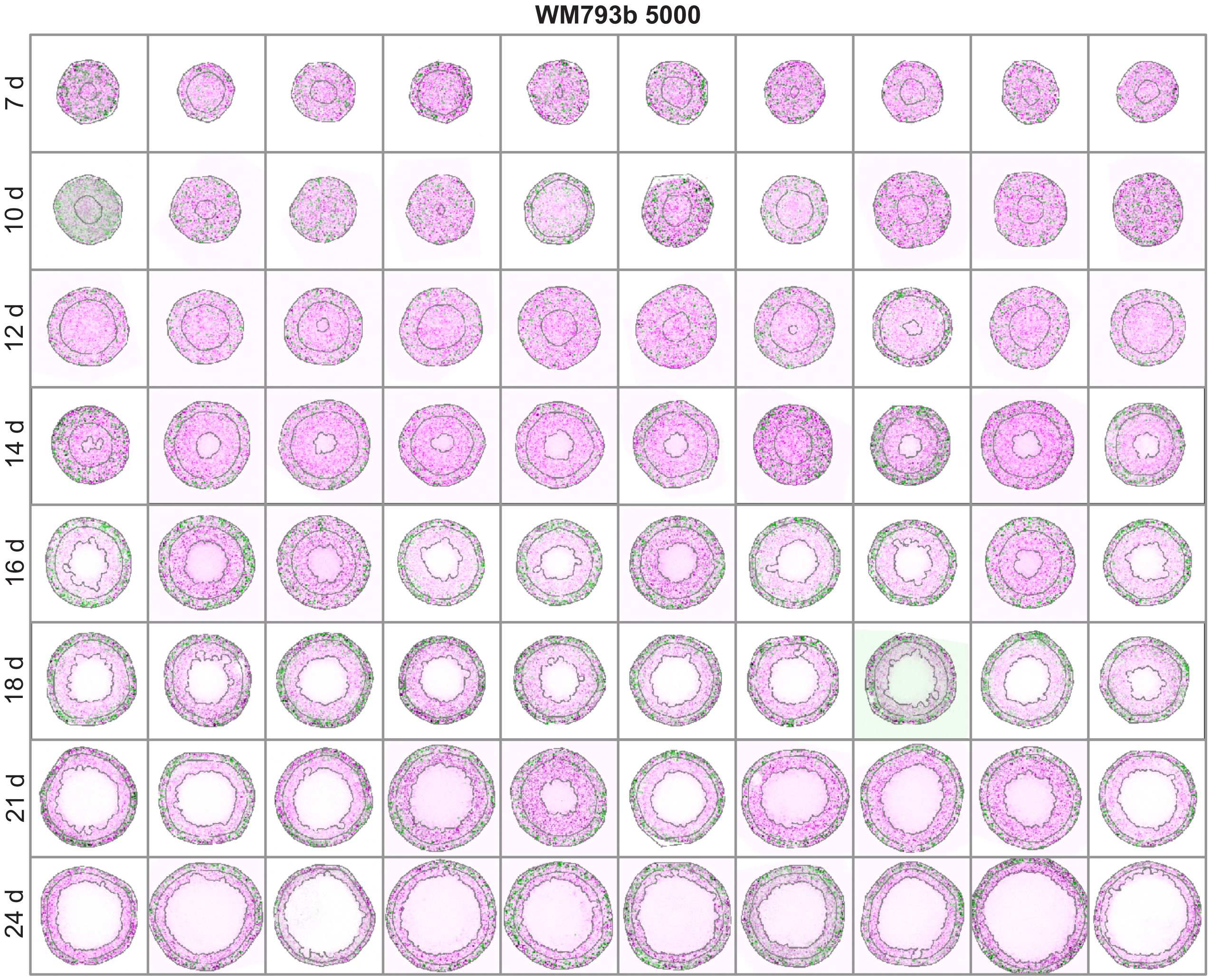

Figure 5

WM793b (seeded with 10000 cells)

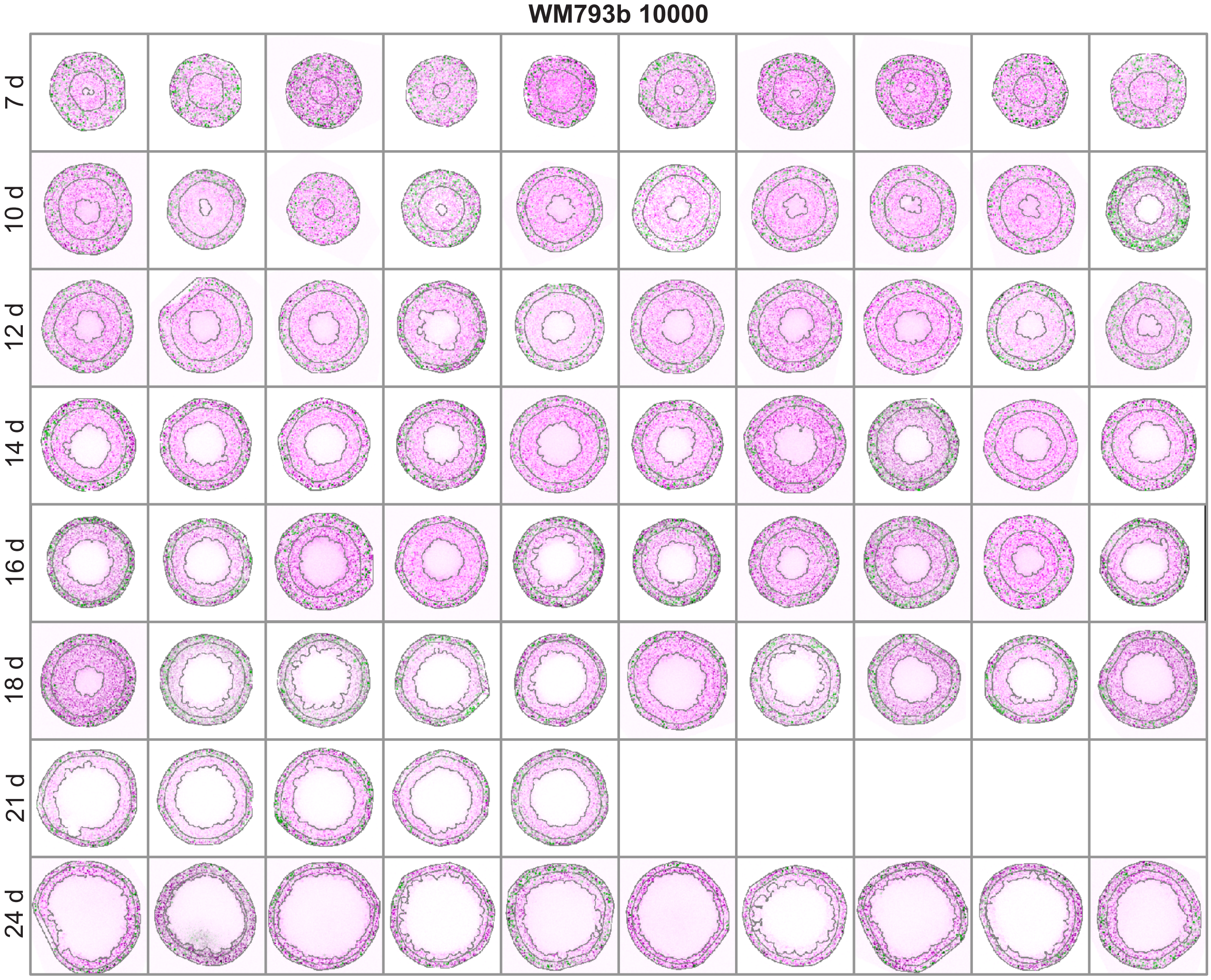

Figure 6
