# Supplementary material for: Quantitative analysis of tumour spheroid structure
Source: eLife. 2021 Nov 29;10:e73020. doi: 10.7554/eLife.73020 (PMC8741212; doi:10.7554/eLife.73020)
Supplement: Supplementary file 3. [file elife-73020-supp3.pdf]

Supplementary file 3

Steady-state analysis for WM983b cells using day 21 data. In the main document, steady-state results in Figure 5 are produced by comparing data at day 21 (spheroids seeded with 2500 cells) to day 18 (spheroids seeded with 5000 and 10000 cells). Here, we reproduce the steady-state results in Figure 5 using data from day 21 from all initial seeding densities. However, we note that between days 18 and 21 spheroids seeded with 5000 and 10000 cells are observed to enter a fourth phase of decay, which is not captured by the mathematical model.

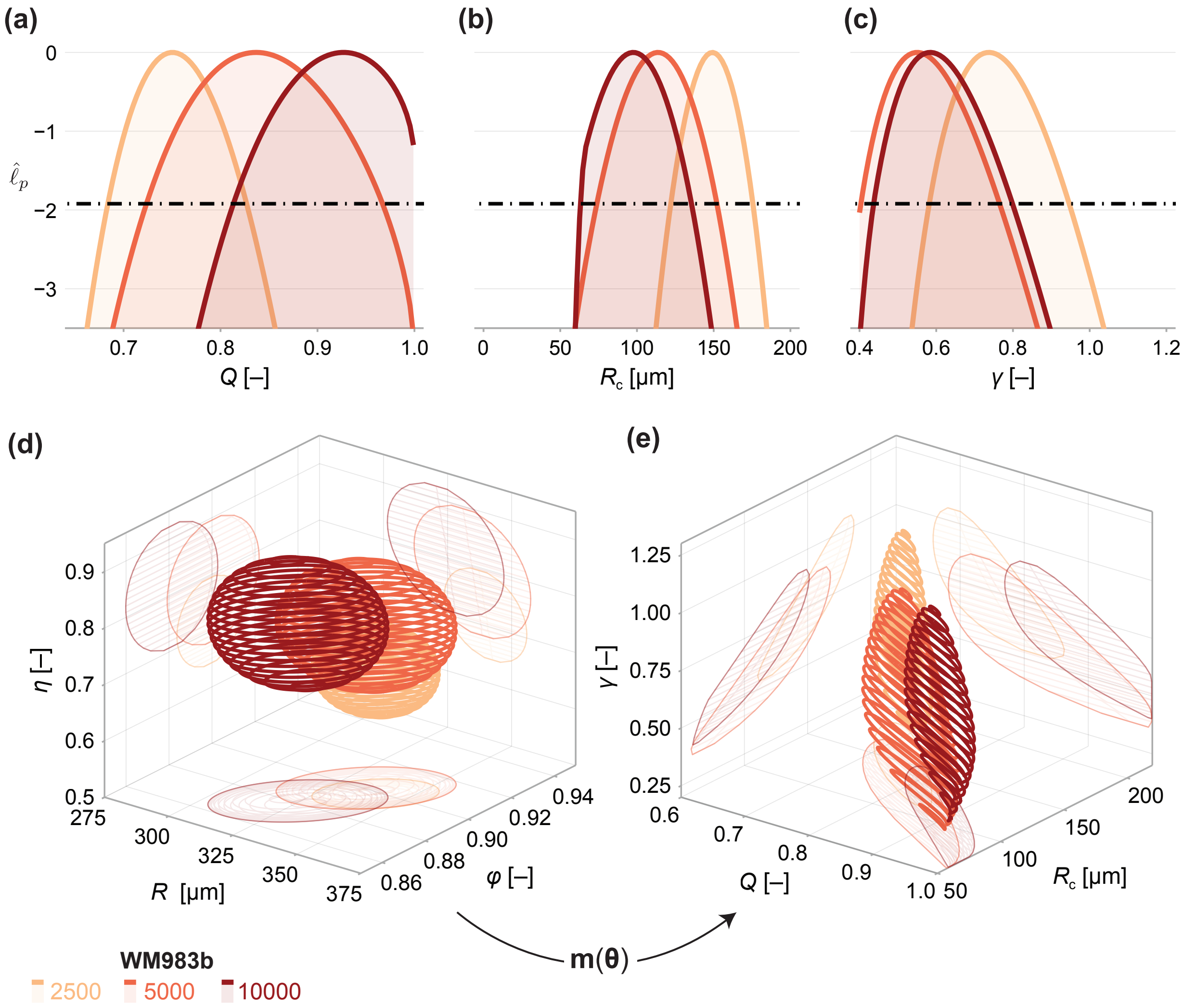

Figure 1. Comparison of WM983b spheroids between each initial seeding density at day 21. (a–c) Profile likelihoods for each parameter. (d) 95% confidence region for the full parameter space. 95% confidence regions for (d) the mean of each observation at steady state $(\bar{R}, \bar{\phi}, \bar{\eta})$ and (e) the model parameters (Q, R_c, γ).
